# Digestive Power of Saliva and Pancreatic Juice during Infancy

**Published:** 1873-11

**Authors:** 


### ARTICLE VIII.

### *Digestive Power of Saliva and Pancreatic Juice during Infancy.*

The recent experiments of Korowin, of St. Petersburg, upon the saliva of newly-born infants and sucklings, in regard to the time of its very earliest appearance and its fermentative power at different ages, well deserve the careful attention of all interested in the dietetics of children. Korowin (*Centralbatt*, 1873, No. 17 and 20) adopted the plan of giving to infants pieces of compressed sponge to suck, and then squeezing from these whatever saliva, if any, might be collected. In this way he was able to determine that saliva may be obtained from the mouth of a child only a few moments born. The secretion is, however, very scanty; indeed, during the first two months of life collecting the saliva is a most difficult process, and demands great perseverence, not more than one cubic centimetre being obtained at one time (fifteen to thirty minutes) in any experiment.

From the end of the first month of life, and especially after the sixth week, the amount of saliva which may be removed increases much. In the third month as much may be obtained as in the first month in one tenth the time. In the fourth month one to one and a half cubic centimetres can be collected in from five to seven minutes, and it is at this age that the saliva begins to flow visibly from the mouth of the child.

The saliva of the child possesses its diastatic or fermentative property from the time of its appearance—that is, immediately after birth. The action of the saliva, however, is not always equally powerful; on the contrary, it increases steadily and rapidly with age up to a certain point, as Korowin was able to determine by watching children for months. It seems certain that while the diastatic power of saliva increases up to the eleventh month of age, it then reaches its maximum—that is, a given amount of saliva of a child of eleven months and of an adult respectively, decomposes equal quantities of starch-paste.

Korowin has also turned his attention to the pancreas and its secretion in newly-born children and in sucklings. The pancreas was removed from the bodies of children who had died of various diseases—at various intervals post-mortem. An artificial pancreatic juice was then prepared in the usual manner, and the amount of glucose formed estimated quantitatively. The results obtained are very important. In a child of one month the action of pancreatic juice upon starch is absolutely *nil*; it is first demonstrable in the second month, but very feeble; at the end of the third month of life it has become sufficiently powerful to make a quantitative estimation possible of the sugar formed. The diastatic action of the pancreatic juice once acquired, steadily increases in intensity with age and reaches its maximum at the end of first year of life.—*Druggists' Circular.*

